# A STING-based fluorescent polarization assay for monitoring activities of cyclic dinucleotide metabolizing enzymes†

**DOI:** 10.1039/d0cb00187b

**Published:** 2020-12-17

**Authors:** Caroline W. Karanja, Kofi S. Yeboah, Wilson W. S. Ong, Herman O. Sintim

**Affiliations:** Department of Chemistry 560 Oval Drive West Lafayette Indiana 47907-2084 USA; Institute for Drug Discovery, Purdue University 720 Clinic Drive West Lafayette IN 47907 USA hsintim@purdue.edu; Purdue Institute of Inflammation, Immunology, and Infectious Disease West Lafayette IN 47907 USA

## Abstract

Cyclic dinucleoties, such as cGAMP, c-di-GMP and c-di-AMP, are fascinating second messengers with diverse roles in both prokaryotes and eukaryotes. Consequently there is a need for simple and inexpensive methods for profiling these compounds in biological media, monitoring their synthesis or degradation by enzymes and for identifying inhibitors of proteins that metabolize or bind to these dinucleotides. Since 2011, when we reported the first simple method to detect c-di-GMP (S. Nakayama, I. Kelsey, J. Wang, K. Roelofs, B. Stefane, Y. Luo, V. T. Lee and H. O. Sintim, *J. Am. Chem. Soc.*, 2011, **133**, 4856) or in 2014 when we revealed another surprisingly simple assay to detect c-di-AMP (J. Zhou, D. A. Sayre, Y. Zheng, H. Szmacinski and H. O. Sintim, *Anal. Chem.*, 2014, **86**, 2412), there have been efforts to develop assays to detect cyclic dinucleotides by others. However a unified and simple assay, which can be used for all cyclic dinucleotides is lacking. Here, we investigate STING binding by various fluorescein-labeled c-di-GMP, c-di-AMP and cGAMP, using fluorescent polarization (FP). Fluorescein-labeled c-di-GMP (F-c-di-GMP) was found to be the best binder of STING. This probe could be displaced by unlabeled cGAMP, c-di-AMP or c-di-GMP and hence it is a universal probe, which can be used to monitor all three dinucleotides. HPLC analysis was used to validate the new F-c-di-GMP-based FP assay.

## Abstract 2

Cyclic dinucleoties, such as cGAMP, c-di-GMP and c-di-AMP, are fascinating second messengers with diverse roles in both prokaryotes and eukaryotes.

## Graphical abstract

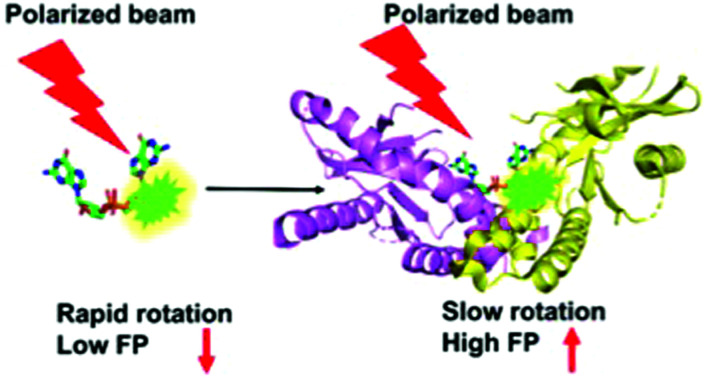

## Introduction

Cyclic dinucleotides (CDNs) have emerged as important signaling molecules in both prokaryotes and eukaryotes and the last decade has witnessed an explosion in research activities related to these fascinating molecules and the enzymes that make or degrade dinucleotides as well as receptors that bind them.^1,2^ Cyclic GMP–AMP synthase (cGAS) and stimulator of interferon genes (STING) are key players in the cGAS–STING pathway, and have emerged as potential drug targets for various disease states, such as viral and bacterial infections, ulcerative colitis, Crohn's disease and cancer.^3–5^ The cGAS–STING pathway in higher organisms, which likely originated in bacteria,^6^ is activated when cytosolic double stranded DNA (pathogen-derived or host-derived) promotes cGAS liquid phase separation and enzyme activity enhancement to produce 2′3-cGAMP (referred to as cGAMP hereafter), which is a noncanonical cyclic dinucleotide containing one 3′-5- and one 2′-5′-phosphodiester linkages.^7–10^ cGAMP binds to dimeric STING, causing profound conformational change and inducing STING aggregation/polymerization, and subsequent TBK1 phosphorylation and activation of IRF3.^11–15^ Interestingly, cGAMP also inhibits STING activation *via* a negative feed-back by triggering ULK1 phosphorylation of STING.^16^ cGAS–STING signaling has been demonstrated to be pivotal for mediation of the innate immune recognition of infected cells and cancer cells.^17–20^ Degradation of cGAMP has emerged as an immune evasion strategy employed by both viral and bacterial pathogens as well as cancer cells.^21–23^ The central roles played by host's cGAMP synthase, cGAS, and cGAMP degrading enzymes (host's ENPP1 (mammalian ectonucleotide pyrophosphate phosphodiesterase 1) or viral poxins) in diseased states have increased interests in finding inhibitors of these enzymes as potential therapeutics. For example patients who harbor TREX1 mutations that lead to loss of DNase activity, and hence are unable to degrade cytosolic DNA (cGAS activator), suffer various inflammatory pathologies.^24,25^ Thus it is expected that inhibitors of cGAS could lessen cGAS–STING pathway activation/inflammation in such patients.^26,27^ Inhibitors of ENPP1 or viral poxins are expected to boost the effects of native cGAMP^28^ and could have applications in cancer immunotherapy^9^ and anti-viral therapy.^8^ c-di-GMP and c-di-AMP are also important second messengers in bacteria, regulating key processes such as biofilm formation, virulence factor production, resistance to antibiotics and metals, amongst many other processes.^1,29^ Thus inhibitors of c-di-GMP and c-di-AMP synthases and phosphodiesterases are also highly sought after as potential new-generation anti-infectives.^30^

**Fig. 1:**
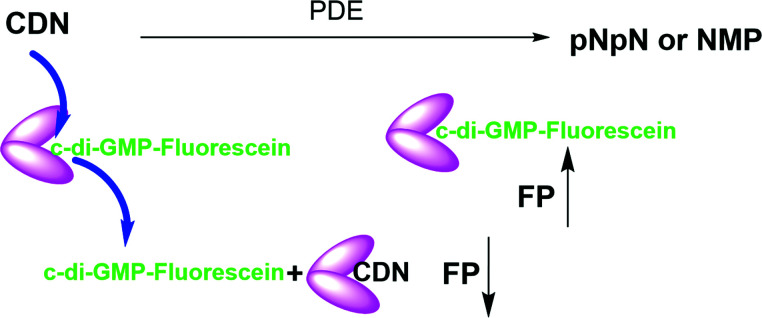
Schematic representation of the working principle of hSTING competitive fluorescence polarization assay. PDE = phosphodiesterase. FP = fluorescence polarization, CDN = cyclic dinucleotide. pNpN = linear dinucleotide. NMP = nucleoside monophosphate. N = nucleoside.

Cheap and reliable assays that could be used to monitor cGAS and cGAMP phosphodiesterases or c-di-GMP/c-di-AMP synthases or phosphodiesterases will undoubtedly facilitate the development of therapeutics that target cGAS-STING signaling in mammalian cells or c-di-GMP/c-di-AMP mediated processes in bacteria. Thus far many strategies have been developed for the detection of cyclic dinucleotides or monitoring dinucleotide metabolism enzymes. For example, radioactive thin-layer chromatography (TLC) has been used to monitor cGAS or ENPP1 activities respectively, but this assay is not convenient due to cost, safety and environmental concerns.^7,31^ Liquid chromatography-mass spectrometry (LC-MS)-based methods can also be used to monitor these enzymes but this method is low throughput. ELISA kits that utilize antibodies to detect cGAMP are commercially available but are expensive. Other methods such as RNA fluorescent c-di-GMP or c-di-AMP or cGAMP sensors,^32–34^ pyrophosphatase-coupled assay,^35^ cGAMP-luciferase assay^36^ and BioSTING assay,^37^ which utilizes FRET, have been described for monitoring cyclic dinucleotides, highlighting the high interests in identifying convenient methods to monitor the aforementioned critical enzymes in the cGAS–STING pathway. Whiles these prior developed assays/biosensors have facilitated CDN research, we found that these methods are not ideal for our medium-to-high throughput screening campaigns for inhibitors of CDN metabolizing enzymes. In the past, we had revealed simple aggregation-based assays for detecting c-di-GMP^38^ or c-di-AMP,^39^ which we have used to identify inhibitors of c-di-AMP synthase *via* medium throughput screening.^40,41^ Unfortunately, our aggregation-based assay is not ideal for monitoring enzymes that degrade cGAMP (our current interest). Thus we sought a more convenient, inexpensive and universal method to monitor the synthesis or degradation of all of the natural cyclic dinucleotides. Herein we disclose a simple method (Fig. 1), which can be used to monitor the degradation or synthesis of any cyclic dinucleotide, which can bind to STING, using readily available fluorescently labeled cyclic dinucleotide probe. We anticipate that others will find this assay useful for their screening campaigns.

## Materials and methods

### hSTING expression and purification

hSTING plasmid was a gift from Prof. Pingwei Li.^42^ hSTING plasmid (PET28a, SUMO) was transformed into Novagen's *E. coli* Rosetta™2(pLysS) cells using kanamycin (50 μg mL^−1^) and chloramphenicol (50 μg mL^−1^) as selection agents. A single colony was then inoculated in 10 mL LB broth supplemented with kanamycin (50 μg mL^−1^) and chloramphenicol (50 μg mL^−1^) followed by an overnight incubation at 37 °C. The next day, the 10 mL culture was inoculated into 1 L terrific broth supplemented with kanamycin (50 μg mL^−1^) and chloramphenicol (50 μg mL^−1^) and grown to exponential phase (OD600 = 0.6) by incubation at 37 °C. 1 mM isopropyl-β-d-thiogalactopyranoside (Fisher Scientific) was added to the culture and temperature lowered to 25 °C to induce protein expression. The culture was incubated at 25 °C for 18 hours. The cells were then pelleted by centrifugation at 5000 rpm for 20 min. The pellet was resuspended in 25 mL lysis buffer containing 50 mM Na_3_PO_4_ (pH = 7.4), 300 mM NaCl, 20 mM imidazole, 5 mM mercaptoethanol, 10% glycerol, 1 mM phenylmethylsulfonyl fluoride and 1× Roche's inhibitor cocktail. The cells were lysed *via* sonication. The lysates were then centrifuged at 22 000 rpm for 25 min and the supernatant collected. The supernatant was passed through a His trap Nickel column and hSTING protein eluted with an elution buffer containing 50 mM Na_3_PO_4_ (pH = 7.4), 300 mM NaCl, 300 mM imidazole, 5 mM mercaptoethanol, and 10% glycerol. hSTING was dialyzed overnight to remove imidazole using a dialysis buffer containing 50 mM Na_3_PO_4_ (pH = 7.4), 300 mM NaCl, 5 mM mercaptoethanol, and 10% glycerol. Protein was quantified by measuring absorbance at 280 nM.

### Poxin expression and purification

Poxin plasmid was a gift from Prof. Philip J. Kranzusch.^21^ The plasmid was transformed into Novagen's *E. coli* Rosetta™2(pLysS) cells using kanamycin (50 μg mL^−1^) and chloramphenicol (50 μg mL^−1^) as selection agents. The enzyme was expressed and purified as described by above. Using 20 mM HEPES–KOH, pH = 7.5, 1 M NaCl, 30 mM imidazole, 10% glycerol and 1 mM DTT as wash buffer, 20 mM HEPES–KOH, pH = 7.5, 400 mM NaCl, 300 mM imidazole, 10% glycerol and 1 mM DTT as elution buffer and 20 mM HEPES–KOH, pH = 7.5, 1 M NaCl, 30 mM imidazole, 10% glycerol and 1 mM DTT as dialysis buffer.

### DisA expression and purification

DisA plasmid was a gift from Prof. Karl-Peter Hopfner.^43^ DisA plasmid (PET28a) was transformed into Novagen's *E. coli* BL21 (DE3) cells using kanamycin (50 μg mL^−1^). The enzyme was expressed and purified as described above. Using 25 mM Tris–HCl, pH = 8.2, 500 mM NaCl, 10% glycerol and 50 mM imidazole as wash buffer, 25 mM Tris–HCl, pH = 8.2, 500 mM NaCl, 10% glycerol and 200 mM imidazole as elution buffer and 25 mM Tris–HCl, pH = 8.2, 500 mM NaCl, 10% glycerol as dialysis buffer.

### WspR expression and purification

WspR plasmid (pVL1394) was a gift from Prof. Md A Motaleb.^44^ The plasmid transformed into Novagen's *E. coli* BL21 (DE3) cells using carbenicillin (100 μg mL^−1^). Wash buffer, elution buffer and dialysis buffer are the same as the buffers used for DisA purification.

### hSTING titration

All four probes were purchased from Biolog Life Science Institute GmbH & Co. KG (Germany). Purity and mass of the probes were confirmed *via* HPLC and mass spectrometry analysis (Fig. S5 and S6, ESI†) F-cGAMP-A = cyclic(guanosine-(2′ → 5±)-monophosphate-2′-*O*-(6-[fluoresceinyl]aminohexylcarbamoyl)adenosine-(3′ → 5′)-monophosphate) sodium salt. F-cGAMP-B = cyclic(8-(2-[fluoresceinyl]aminoethylthio)-guanosine-(2′ → 5′)-monophosphate-adenosine-(3′ → 5′)-monophosphate) (c[8-Fluo-AET-G(2′,5′)pA(3′,5′)p]), sodium salt. F-c-di-GMP = 2′-*O*-(6-[fluoresceinyl]aminohexylcarbamoyl)-cyclic diguanosine monophosphate (2′-Fluo-AHC-c-diGMP), sodium salt. F-c-di-AMP = 2′-*O*-(6-[fluoresceinyl]aminohexylcarbamoyl)-cyclic diadenosine monophosphate (2′-Fluo-AHC-c-diAMP), sodium salt.

50 nM probe was incubated with various concentrations of hSTING in 1× phosphate buffered saline for 5 min at room temperature and fluorescence polarization (excitation 485 nm/20 and emission 528 nm/20) detected with Biotek Cytation 5 multi-mode reader. Anisotropy was calculated using the Gen 5™ microplate reader and imaging software. Anisotropy was normalized by equating measurements of the 0 μM hSTING group to zero. Experiment was done in triplicates using a 384 fluorometric plate.

### Probes displacement assays

50 nM F-c-di-GMP was incubated with 10 μM hSTING and different concentrations of cGAMP for 5 min at room temperature and Anisotropy determined as described above. Anisotropy was normalized by equating measurements of 0 μM cGAMP group to 100. Experiment was done in triplicates using a 384 fluorometric plate.

### ENPP1 cGAMP hydrolysis tracking

A reaction was set up containing 50 μM cGAMP, and 9.16 nM hENPP1 (purchased from R&D Systems (Minneapolis, Minnesota)) using the reaction buffer recommended by the manufacturer (50 mM Tris pH 9.5, and 250 mM NaCl). At specific time points, 90 μL was drawn from the reaction, reaction stopped by heat denaturation and cGAMP concentration detected *via* the FP assay and HPLC analysis. For FP detection, 35 μL of the reaction was mixed with 10 μM hSTING, 50 nM F-c-di-GMP and topped to 70 μL with 1× phosphate buffered saline. The Mix was incubated at room temperature for 5 min. 20 μL of the mix was aliquoted into each well of the 384 fluorometric plate and anisotropy determined as described above. Anisotropy was normalized by equating measurements at time zero group to zero. Experiment was done in triplicates using a 384 fluorometric plate. 50 μL of the reaction was subjected to HPLC analysis using a COSMOSIL C18-MS-II Packed column (mobile phase = 0.1 M TEAA in water and acetonitrile). Gradient is as follows: 0–16 min: 99%–87% 0.1 M TEAA, 1–13% acetonitrile, 16–23 min: 87–10% 0.1 M TEAA, 13–90% acetonitrile, 23–25 min: 10–99% 0.1 M TEAA, 90–1% acetonitrile nucleotides were detected by measuring absorbance at 260 nm.

### Poxin cGAMP hydrolysis tracking

A reaction was set up containing 50 μM cGAMP, and 5 nM poxin using the reaction buffer (50 mM HEPES–KOH pH 7.5, 35 mM KCl, and 1 mM DTT). At specific time points, 90 μL was drawn from the reaction, reaction stopped by heat denaturation and cGAMP concentration detected *via* the FP assay and HPLC analysis. FP detection and HPLC analysis were conducted as described above.

### WspR c-di-GMP synthesis detection with FP assay

A reaction was set up containing 100 μM GTP, and 11 μM WspR in WspR buffer (10 mM Tris–HCl pH 7.5, 100 mM Nacl, and 5 mM MgCl_2_) and incubated at 37 °C for 16 h. The reaction was stopped by incubating at 95 °C for 5 min. c-di-GMP synthesis was detected using 10 μM hSTING and 50 nM F-c-di-GMP in a 96 well fluorometric plate.

### WspR c-di-GMP synthesis detection with HPLC

A reaction was set up containing 100 μM GTP, and 11 μM WspR in WspR buffer (10 mM Tris–HCl pH 7.5, 100 mM NaCl, and 5 mM MgCl_2_) and incubated at 37 °C for 16 h. The reaction was stopped by incubating at 95 °C for 5 min and centrifuged at 14 000 rpm for 3 min. 100 μL of reaction was filtered in an ultrafree centrifuge filter and subjected to the HPLC analysis. Conditions for HPLC detection was the same as described in the hydrolysis tracking of ENPP1.

### DisA c-di-AMP synthesis detection with FP assay

A reaction was set up containing 100 μM ATP, 1 μM DisA in DisA buffer (40 mM Tris–HCl pH 7.5, 100 mM NaCl, and 10 mM MgCl_2_) and incubated at 37 °C for 16 h. FP detection was done as described for WspR.

### DisA c-di-AMP synthesis detection with HPLC

A reaction was set up containing 100 μM ATP, and 1 μM DisA in DisA buffer (40 mM Tris–HCl pH 7.5, 100 mM NaCl, and 10 mM MgCl_2_) and incubated at 37 °C for 16 h. HPLC detection conditions for DisA was the same as for WspR.

### Emmission spectrum of fluorescent probes

F-cGAMP-A, F-cGAMP-B, F-c-di-GMP and F-c-di-AMP at a concentration of 12.5 nM in 200 μL 1× phosphate-buffered saline (PBS) was used for the emission spectra. The excitation was done at 480 nm and emission was collected from 510 nm to 600 nm.

### Data analysis

Curves were generated with origin software using in-built nonlinear functions or stimulated with eqn (3).

## Results and discussion

### F-c-di-GMP is an ideal probe for hSTING FP assay

STING has a high affinity for cyclic dinucleotides and secondly fluorescence polarization has been demonstrated to be a robust technique to probe protein–ligand interactions.^45–47^ Thus we rationalized that an appropriately fluorescent-labeled cyclic dinucleotide could be used to monitor the presence of unlabeled cGAMP *via* competition for STING binding (as shown in Fig. 1). STING can be readily expressed in *E. coli*,^12^ so a fluorogenic displacement assay using STING could be cheaper to perform than previously described assays for detecting cyclic dinucleotides (such as cGAMP), which use monoclonal antibodies.^48^ Additionally, since STING can also bind to other cyclic dinucleotides (c-di-GMP and c-di-AMP), we reasoned that a STING-based fluorescent polarization assay could be readily adapted to monitor c-di-GMP and c-di-AMP metabolizing enzymes whereas the monoclonal antibody approach would generally require specific antibodies for each dinucleotide. The working principle of the assay is illustrated in Fig. 1. In the presence of STING binding cyclic dinucleotides the fluorescence polarization of the probe is low owing to the fact unlabeled cyclic dinucleotide displaces the probe from hSTING. However, following the addition of a cyclic dinucleotide degrading enzyme such as ENPP1 or viral poxin, there would be an increase in the fluorescence polarization as time progresses. As time progresses, the unlabeled cyclic dinucleotide is degraded and concentration decreases allowing the labeled probe to bind STING leading to increase in fluorescence polarization. The converse will be true for a cyclic dinucleotide synthase, which would increase the amount of cGAMP or c-di-GMP or c-di-AMP, which would compete with the fluorescent probe to decrease signal. Thus the assay is a universal one for monitoring enzymatic dynamics of either CDN synthase or phosphodiesterase.

**Fig. 2:**
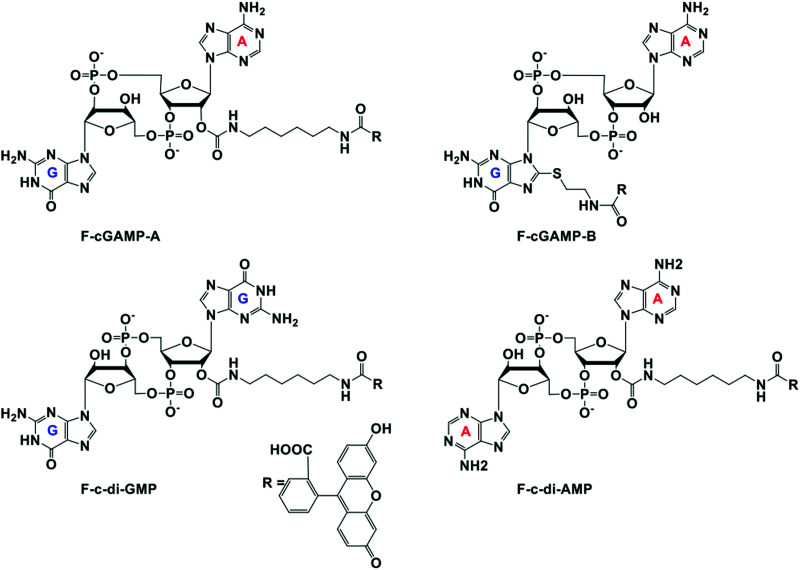
Structures of fluorescein labeled cyclic dinucleotide used in this work.

At the onset of the project, it was not clear how modification with a fluorophore would affect STING binding. The binding constants for the natural cyclic dinucleotides for hSTING are: *K*_d_ for cGAMP is 3.79 nM; *K*_d_ for c-di-GMP is 1210 nM and *K*_d_ for c-di-AMP is 1382 nM.^9^ Because cGAMP has an ultra-potent affinity for STING, we rationalized that even if modification reduced binding affinity, there could still be some affinity for STING left for practical detection. Thus, we selected two labeled cGAMP probes, where the fluorescein modifications were at the 2′-position of adenosine (F-cGAMP-A) and at the C8-position of guanosine (F-cGAMP-B) (Fig. 2). We were however aware that the X-ray crystal structure of cGAMP bound to STING indicates that the ligand makes intimate contacts with the protein residues and also the ligand is bound in the closed conformation whereby cGAMP is engulfed by the protein.^11,49^ Therefore there was a high probability that any modification of cGAMP would drastically reduce STING binding. c-di-GMP or c-di-AMP on the other hand bind to STING in the open conformation and we hypothesized that c-di-GMP or c-di-AMP would better tolerate fluorescein modification at the 2′-OH (see F-c-di-GMP and F-di-AMP, Fig. 2) than any modifications to cGAMP.^50,51^ This hypothesis was supported by literature precedence, whereby Wu *et al.* showed that c-di-GMP that was modified at the 2′-OH position with a bulky glycopeptide could still activate STING.^52^

**Fig. 3:**
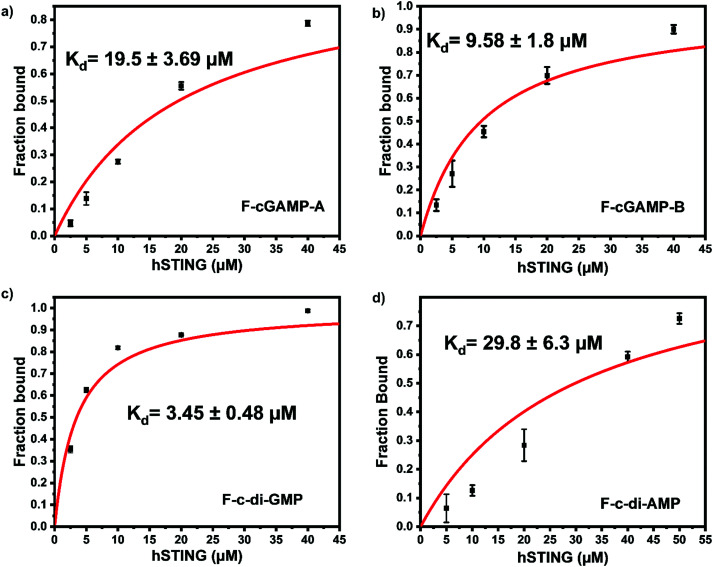
F-c-di-GMP is an ideal probe for hSTING fluorescence polarization competitive assay. Fraction bound *versus* concentration of STING of (a) F-cGAMP-A; (b) F-cGAMP-B; (c) F-c-di-GMP; (d) F-c-di-AMP. Error bars represents the standard deviation of *n* = 3 replicates.

To identify the ideal probe for the assay, we investigated which of the four fluorescein labeled cyclic dinucleotide probes bound hSTING best by titrating each probe (50 nM) with different concentrations of hSTING and measuring fluorescence polarization (FP). Fluorescence polarization measurements were then converted into fluorescence anisotropy using eqn (1).^53^1
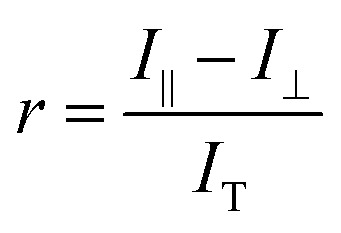
Where *I*_‖_ is the intensity of the parallel emission, *I*_⊥_ is the intensity of the perpendicular emission and *I*_T_ is total intensity. To rank the binding affinities of the probes, anisotropy was converted to fraction bound (eqn (2))^54^ and the dissociation constant (*K*_d_) of each probe evaluated.2
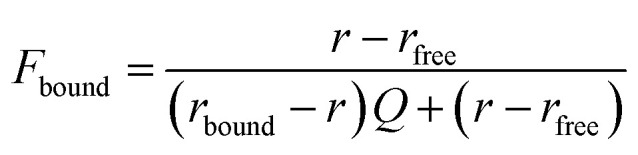
Where *r* is anisotropy at a specific hSTING concentration, *r*_free_ is anisotropy of free ligand, *r*_bound_ is the anisotropy of hSTING-probe at saturation and *Q* is the ratio of fluorescence intensities of bound *versus* free ligand. Fraction bound *versus* hSTING concentration curves were generated using eqn (3) (Fig. 3a–d).^55,56^3
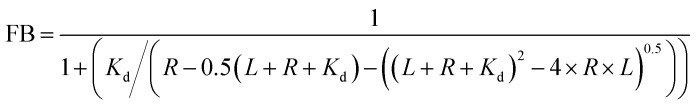
Where FB is fraction bound, *K*_d_ is the dissociation constant, *R* is hSTING concentration and *L* is the probe concentration.

Despite possessing the lowest emission intensity, F-c-di-GMP is the best hSTING binder with a *K*_d_ of 3.45 ± 0.48 μM, whiles F-c-di-AMP is the weakest hSTING binder with a *K*_d_ of 29.8 ± 6.3 μM (Fig. 3 and ESI,† Fig. S1, S2). In spite of the fact that F-cGAMP-B binds hSTING slightly better than F-cGAMP-A, F-cGAMP-A exhibits a higher signal range than F-cGAMP-B. High anisotropy is observed in the F-cGAMP-A group compared to F-cGAMP-B (ESI,† Fig. S1a and b). This phenomenon is likely due to guanine quenching the fluorophore in F-cGAMP-B, which is attached to guanine *via* a short linker; guanine is a known fluorescence quencher.^57^ Expectedly, F-cGAMP-B emission intensity is lower than that of F-cGAMP-A (ESI,† Fig. S2).

**Fig. 4:**
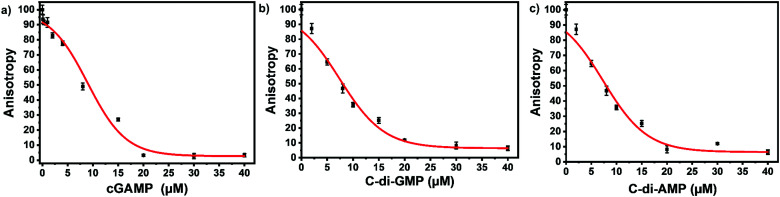
Displacement of F-c-di-GMP with unlabeled cyclic dinucleotides. (a) Displacement using cGAMP. (b) Displacement using c-di-GMP (c) displacement using c-di-AMP. 50 nM F-c-di-GMP and 10 μM hSTING used for all three displacement experiments. Curves generated with origin in-built dose response function.

We picked F-c-di-GMP as our ideal probe since it has the highest binding affinity to STING, compared to the other fluorescein-labeled probes. Next, we sought to determine if unlabeled cyclic dinucleotides could displace F-c-di-GMP from hSTING. Displacement of the probes from STING would lead to a decline in anisotropy, brought about by the increase in the concentration of free fluorescent ligands. As expected unlabeled cyclic dinucleotides displaced F-c-di-GMP in a concentration dependent manner with apparent half maximal inhibitory concentrations of 8.95 ± 0.54 μM (cGAMP), 7.41 ± 0.49 μM (c-di-GMP), and 7.35 ± 0.86 μM (c-di-AMP) (Fig. 4a–c). These findings illustrate our assay could be used to track enzymatic dynamics of cyclic dinucleotide metabolizing enzymes. Consequently, we proceeded to investigate if our assay could track the progresses of cGAMP degradation by two enzymes: ENPP1 and poxin.

### Monitoring ENPP1 hydrolysis of cGAMP with hSTING competitive assay

**Fig. 5:**
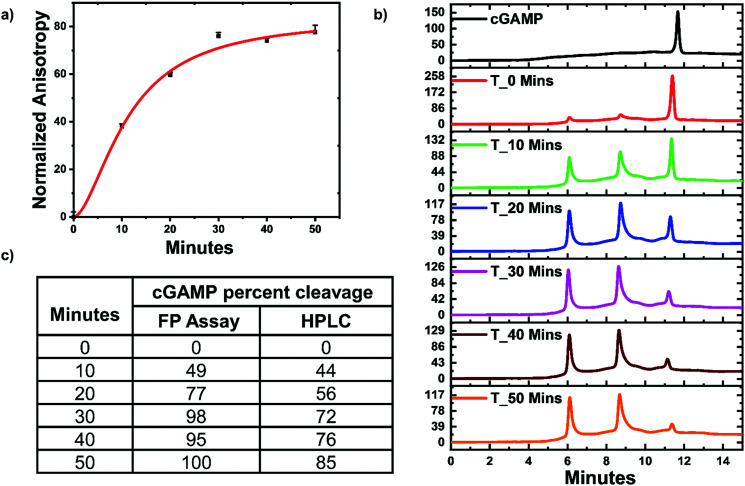
hSTING fluorescence polarization competitive assay can track ENPP1 cGAMP hydrolysis. (a) Time trace anisotropy of ENPP1 reactions. Curve generated with origin in-built Hill function. (b) HPLC time trace of ENPP1 reactions. (c) cGAMP percent cleavage at specific time points quantified with either FP assay or HPLC analysis. cGAMP percent cleavage was calculated by assuming anisotropy after 50 min represented 100% cleavage for the FP assay. For HPLC analysis, peak area of cGAMP at time zero was used to normalize cGAMP signal in all the time points then percent cleavage was computed by subtracting the normalized peak area from 100.

ENPP1 hydrolyzes cGAMP into GMP and AMP, and both do not bind hSTING. Hence, we hypothesized that our assay could be used to monitor ENPP1 hydrolysis of cGAMP. We conducted a time trace experiment whereby ENPP1 reactions were stopped by heat denaturation after a specific time and cGAMP concentration detected with the FP assay and *via* liquid chromatography analysis (HPLC). Since ENPP1 cGAMP hydrolysis end products, AMP and GMP, do not bind to hSTING fluorescence anisotropy is expected to increase as a function of time. As expected, anisotropy increased with time (Fig. 5a). More importantly, the assay correlated with the HPLC analysis (Fig. 5b and c). At time zero, anisotropy is almost zero indicating probe is not binding to hSTING due to the high concentration of unlabeled cGAMP as evident in the HPLC trace for time zero (Fig. 5a and b). As time progress, cGAMP is hydrolyzed by ENPP1, which leads to an increase in FP and decrease of the cGAMP peak in the HPLC traces. After 30 min, there is no significant change in anisotropy, an indication that most of the cGAMP is hydrolyzed (Fig. 5a). This observation roughly correlates with the HPLC traces, which also indicate that over 70% of the reaction cGAMP is hydrolyzed within 30 min (Fig. 5).

### Monitoring poxin hydrolysis of cGAMP with hSTING competitive assay

**Fig. 6:**
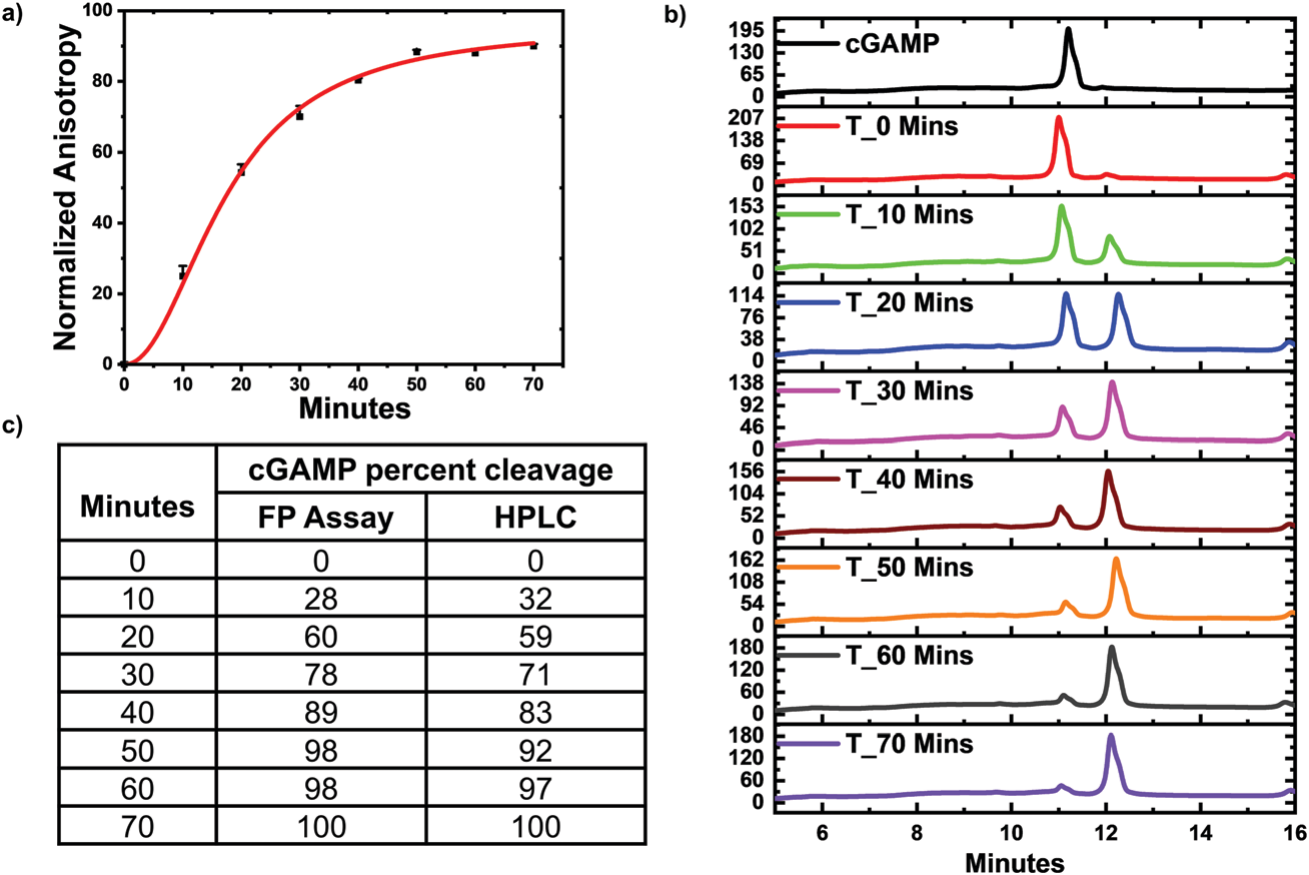
hSTING fluorescence polarization competitive assay can track poxin cGAMP hydrolysis. (a) Time trace anisotropy of poxin reactions. Curve generated with origin in-built Hill function. (b) HPLC time traces of poxin reactions. (c) cGAMP percent cleavage at specific time points quantified with either FP assay or HPLC analysis. cGAMP percent cleavage was calculated by assuming anisotropy after 70 min represented 100% cleavage for the FP assay. For HPLC analysis, peak area of poxin product at 70 min was assumed to represent 100% cleavage.

Poxin is a metal-independent nuclease conserved in most *Orthopoxvirus* viruses that cleaves cGAMP to inhibit hSTING signaling.^21^ Poxin degrades cGAMP into linear Gp[2′–5′]Ap[3′], which does not bind hSTING.^21^ Thus poxin cleavage of cGAMP could also be monitored with hSTING competitive fluorescence polarization assay. To test this, poxin reactions were set up and after a specific time the reaction was stopped by heat denaturation and cGAMP concentration detected with our assay and *via* HPLC analysis. Just as in the case of ENPP1, the assay trend agreed with the HPLC analysis. Both the hSTING assay and the HPLC show poxin degraded most of the cGAMP within 50 min (Fig. 6). After 50 min, there is no significant change in the anisotropy measurements (Fig. 6a and c). Similarly, there is no significant change in both cGAMP and poxin product peaks after 50 min as visualized in the HPLC traces (Fig. 6b).

### Monitoring the synthesis of c-di-GMP and c-di-AMP with hSTING competitive assay

**Fig. 7:**
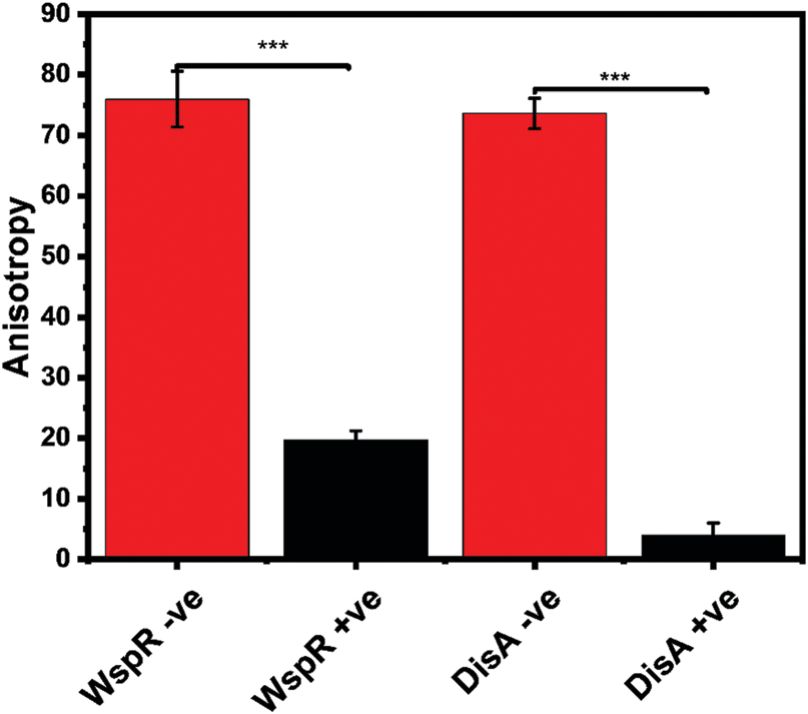
hSTING fluorescence polarization competitive assay can track WspR and DisA synthesis dynamics. WspR reactions: 11 μM enzyme and 100 μM GTP in 1× reaction buffer (10 mM Tris–HCl pH 7.5, 100 mM NaCl and 5 mM MgCl_2_) and incubate at 37 °C for 16 h. WspR −ve = Reactions containing no WspR. WspR +ve = Reactions containing WspR. DisA reactions: 1 μM enzyme and 100 μM ATP in 1× reaction buffer (40 mM Tris–HCl pH 7.5, 100 mM NaCl and 10 mM MgCl_2_) and incubate at 37 °C for 16 h. DisA −ve = Reactions containing no DisA. DisA +ve = Reactions containing DisA. Error bars represent standard deviation of *n* = 3.

Our group is also interested in identifying compounds that inhibit c-di-GMP or c-di-AMP synthesis in bacteria. Thus, we sought to determine if the FP assay could also track the synthesis of c-di-AMP or c-di-GMP. We set up reactions with WspR (c-di-GMP synthase)^58^ and DisA (c-di-AMP synthase)^43^ and probed them with the FP hSTING competitive assay and with HPLC. After incubation, reactions were stopped by heat denaturing and half of the sample was analyzed with the hSTING assay and the other half subjected to HPLC analysis. As expected, due to presence of c-di-AMP or c-di-GMP, anisotropy was significantly lower for reactions containing both WspR and DisA compared to reactions that did not contain the synthases (Fig. 7). To confirm the low anisotropy was indeed a result of displacement by unlabeled c-di-AMP or c-di-GMP, we obtained HPLC traces of both the DisA and WspR reactions. Both enzymes synthesized each respective cyclic dinucleotide (ESI,† Fig. S3a, b and S4a, b).

## Conclusion

Cyclic dinucleotides have come to the forefront of biological research due to the central roles they play in various physiological processes in both bacteria and metazoans.^59^ Enzymes and adaptor proteins that sense and/or regulate these second messengers are now *bona fide* drug targets and there is a need for simpler and cheaper assays to detect these molecules for various applications.^60^ In 2011 we provided one of the earliest detections of cyclic dinucleotides using homogeneous aggregation strategy.^38^ In 2012, we reported the first RNA-based detection of cyclic dinucleotides.^34^ Since then various strategies have been described to detect cyclic dinucleotides.^33,61–65^ We have since been looking for a universal and simple assay that could be used to detect all of the dinucleotides. Here, we present a simple assay that can facilitate cyclic dinucleotide research as well as provide a platform to discover compounds, which will translate the beautiful biology of cyclic dinucleotides into real life therapeutics. Although we have used a few enzymes, ENPP1, poxin, WspR and DisA, to demonstrate this concept in the manuscript, there is no conceptual impediment preventing the adaptation of this assay to detect any cyclic dinucleotide metabolism enzymes. This newly developed assay has streamlined our work evaluating cyclic dinucleotide metabolism enzymes and we hope that others will also find the described assay useful.

## Conflicts of interest

There are no conflicts to declare. 

## Supplementary Material

CB-002-D0CB00187B-s001
