# Supplementary material for: A STING-based fluorescent polarization assay for monitoring activities of cyclic dinucleotide metabolizing enzymes
Source: RSC Chem Biol. 2020 Dec 17;2(1):206–14. doi: 10.1039/d0cb00187b (PMC8341399; doi:10.1039/d0cb00187b)
Supplement: CB-002-D0CB00187B-s001 [file CB-002-D0CB00187B-s001.pdf]

Electronic Supplementary Material (ESI) for RSC Chemical Biology.
This journal is © The Royal Society of Chemistry 2020

A STING-based Fluorescent Polarization Assay for Monitoring Activities of Cyclic Dinucleotide Metabolizing Enzymes

Caroline W. Karanja,[a] Kofi S. Yeboah,[a] Wilson W.S. Ong[a] and Herman O. Sintim[a,b,c*]

[a] Department of Chemistry, 560 Oval Drive, West Lafayette, Indiana 47907-2084
[b] Institute for Drug Discovery, Purdue University, 720 Clinic Drive, West Lafayette, IN 47907, USA.
[c] Purdue Institute of Inflammation, Immunology, and Infectious Disease, West Lafayette, IN, 47907, USA.
*Corresponding author. Email: hsintim@purdue.edu

Supplementary information

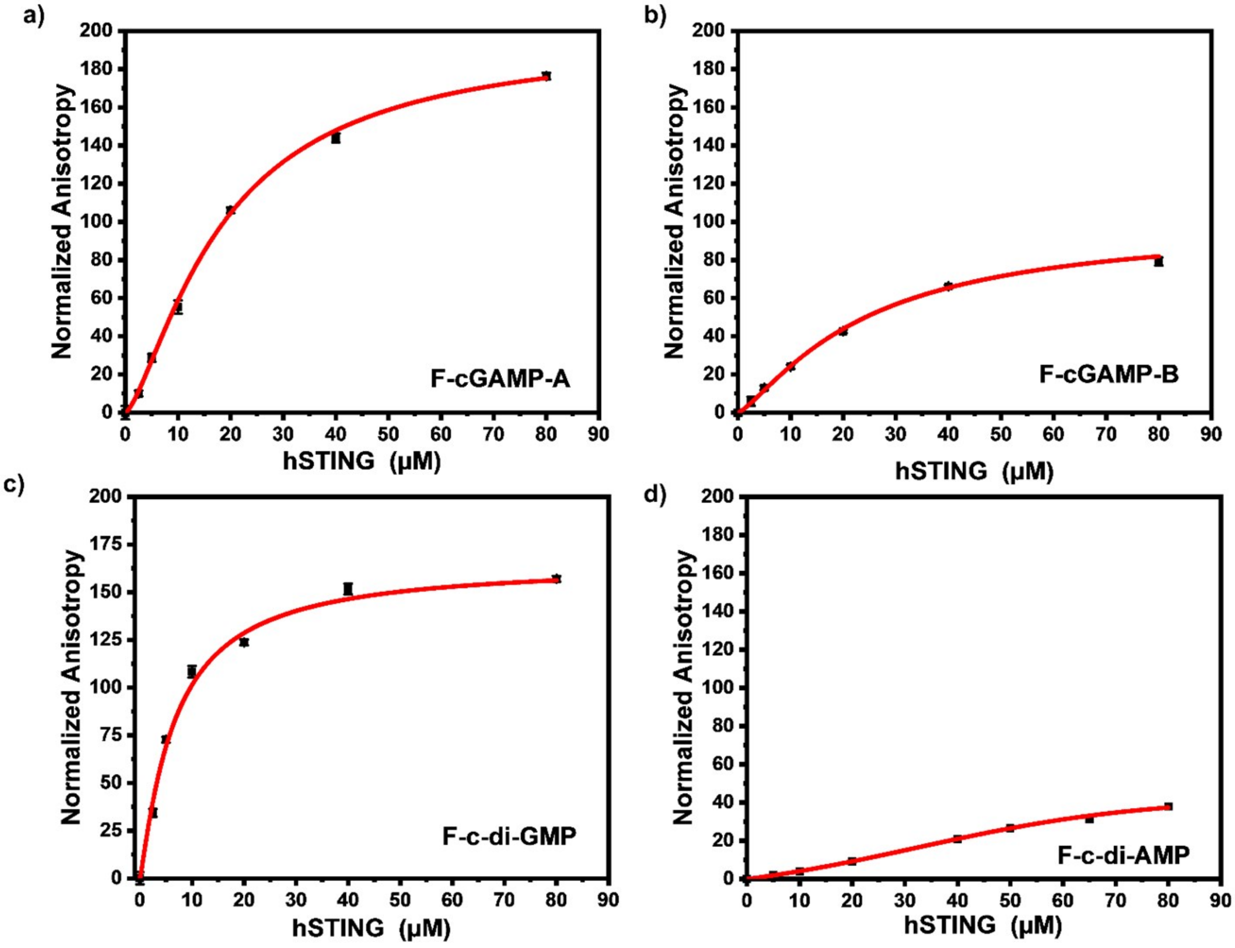

Fig. S1: hSTING titration curves a) Anisotropy of F-cGAMP-A in the presence of different concentrations of hSTING. b) Anisotropy of F-cGAMP-B in the presence of different concentrations of hSTING. c) Anisotropy of F-c-di-GMP in the presence of different concentrations of hSTING. d) Anisotropy of F-c-di-AMP in the presence of different concentrations of hSTING. Curves generated with origin in-built dose response function.

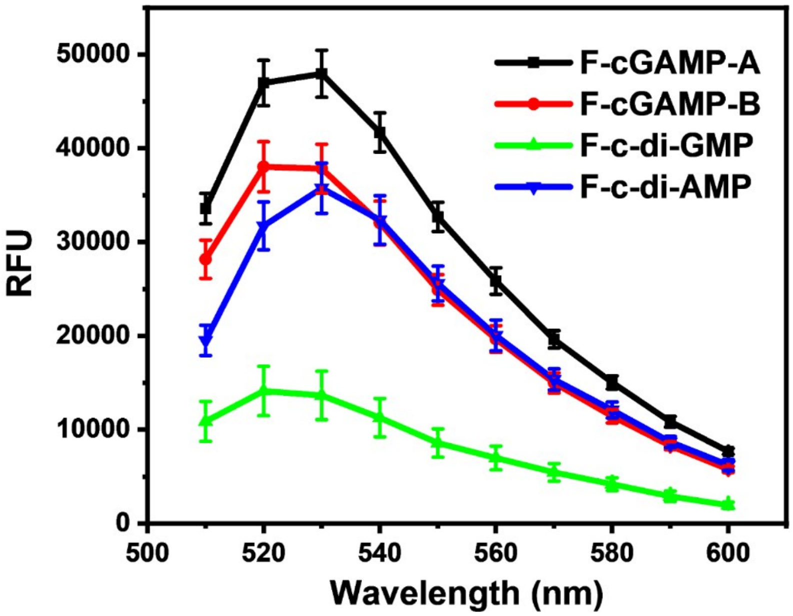

Fig. S2: F-cGAMP-A, F-cGAMP-B, F-c-di-GMP and F-c-di-AMP emission spectra. Emission spectra generated using 12.5 nM probe in 1x PBS. Excitation: 480/20 nm. Error bars represent standard deviation of n=3.

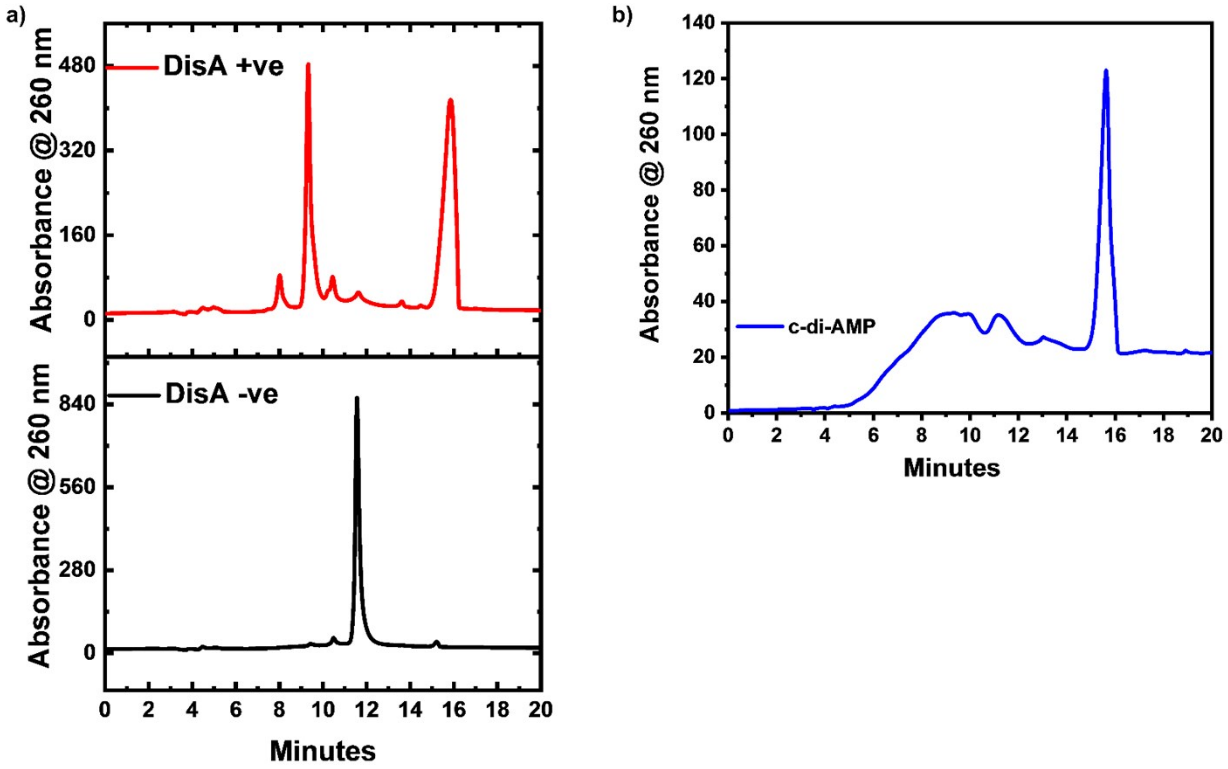

Fig. S3: HPLC traces of DisA reactions. a) HPLC traces of DisA +ve and DisA -ve reactions. DisA reactions: 1 µM enzyme and 100 µM ATP in 1x reaction buffer (40 mM Tris-HCl pH 7.5, 100 mM NaCl, and 10mM $MgCl_2$) and incubate at 37 ^{o}C for 16 h. DisA –ve = Reactions containing no DisA. DisA +ve = Reactions containing DisA. b) HPLC trace of c-di-AMP standard.

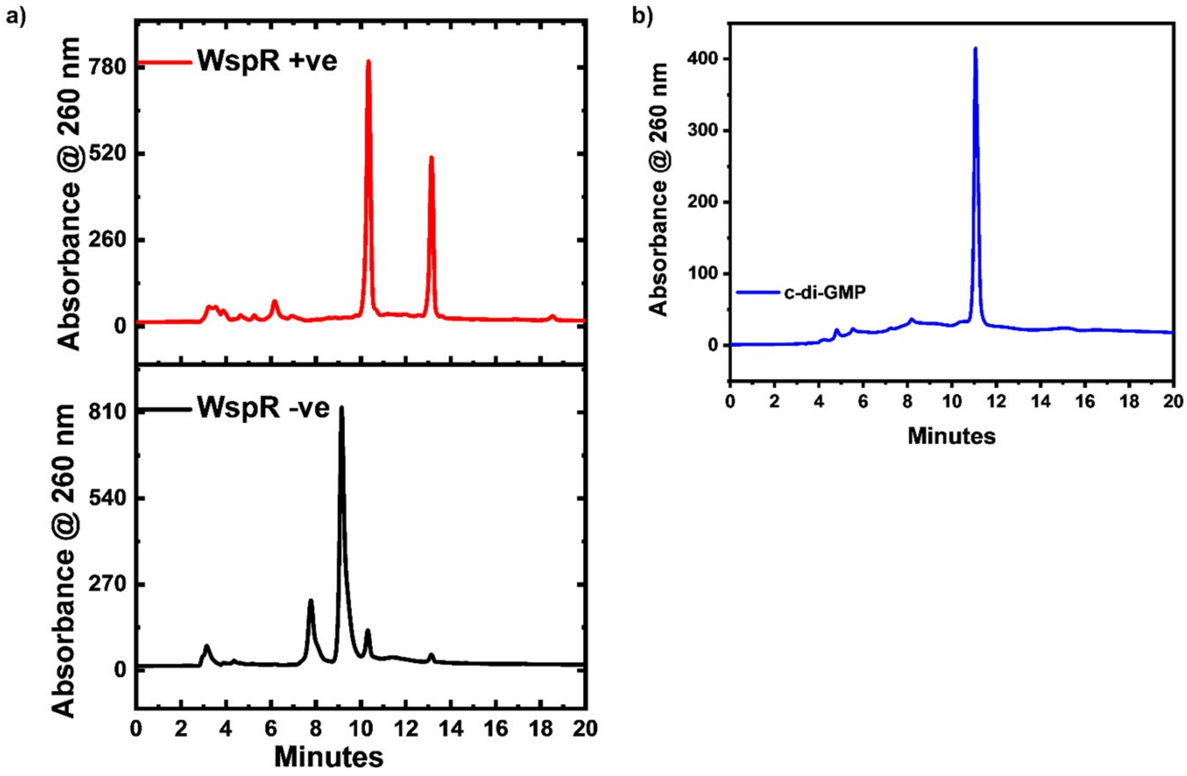

Fig. S4: HPLC traces of WspR reactions. a) HPLC traces of WspR +ve and WspR -ve reactions. WspR reactions: 11 µM enzyme and 100 µM GTP in with 1x reaction buffer (10 mM Tris-HCl pH 7.5, 100 mM NaCl, and 5mM $MgCl_2$) and incubate at 37 ^{o}C for 16 h. WspR –ve = Reactions containing no WspR. WspR +ve = Reactions containing WspR. b) HPLC trace of c-di-GMP standard.

Purity analysis of fluorescent cyclic dinucleotides via HPLC. Fluorescent cyclic -dinucleotides were dissolved in water to yield a 100 µM solution, and 100 µL of the solution was subjected to HPLC analysis using a AGILENT Eclipse Plus C18- Packed column (particle size 3.5µm; 4.6 x 100mm) (mobile phase = water and acetonitrile). Gradient is as follows: 0 – 16 min: 99 % - 87 % water, 1% -13% acetonitrile, 16 – 23 min: 87 % - 10 % water, 13 % - 90 % acetonitrile, 23 – 25 min: 10 % - 99 % water, 90 % -1 % acetonitrile. Nucleotides were detected by measuring absorbance at 260 nm.

Mass analysis of fluorescent cyclic dinucleotides via MALDI-TOF MS. Fluorescent cyclic dinucleotides were dissolved to yield a 10 µM solution and was desalted prior to analysis using ZipTip pipette tips. A 10 mg/mL solution of α-cyano-4-hydroxycinnamic acid in a 1:1 mixture of acetonitrile and 0.1% trifluoroacetic acid in water was used as the matrix. Aliquots of the desalted sample were combined with an equal volume of the matrix and transferred in 1 µL quantities onto a 400-well MALDI plate. MALDI-TOF/TOF spectra was obtained using a 4800 Proteomics Analyzer (Applied Biosystems). Spectra were acquired in the positive ion mode.

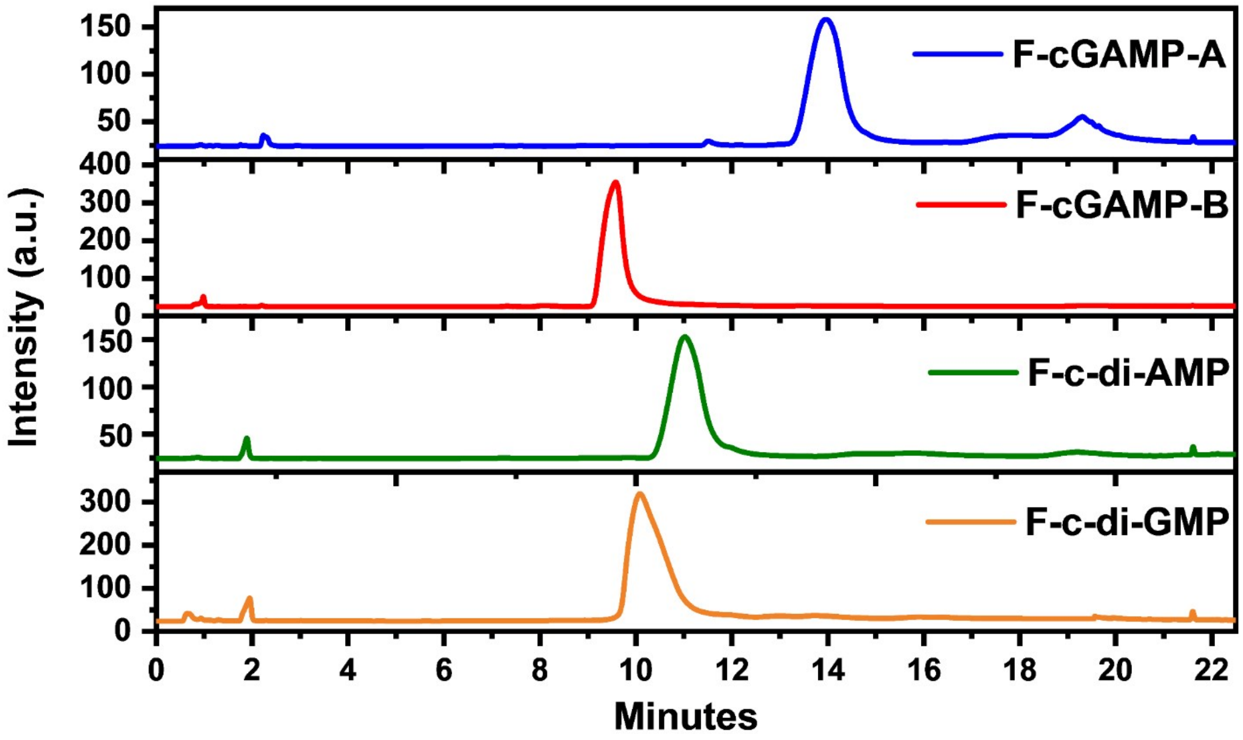

Fig. S5: HPLC traces of Fluorescent cyclic dinucleotides used for the fluorescence polarization assay. Fluorescent cyclic dinucleotides used have a purity of >90% as quantified by HPLC analysis.

a)

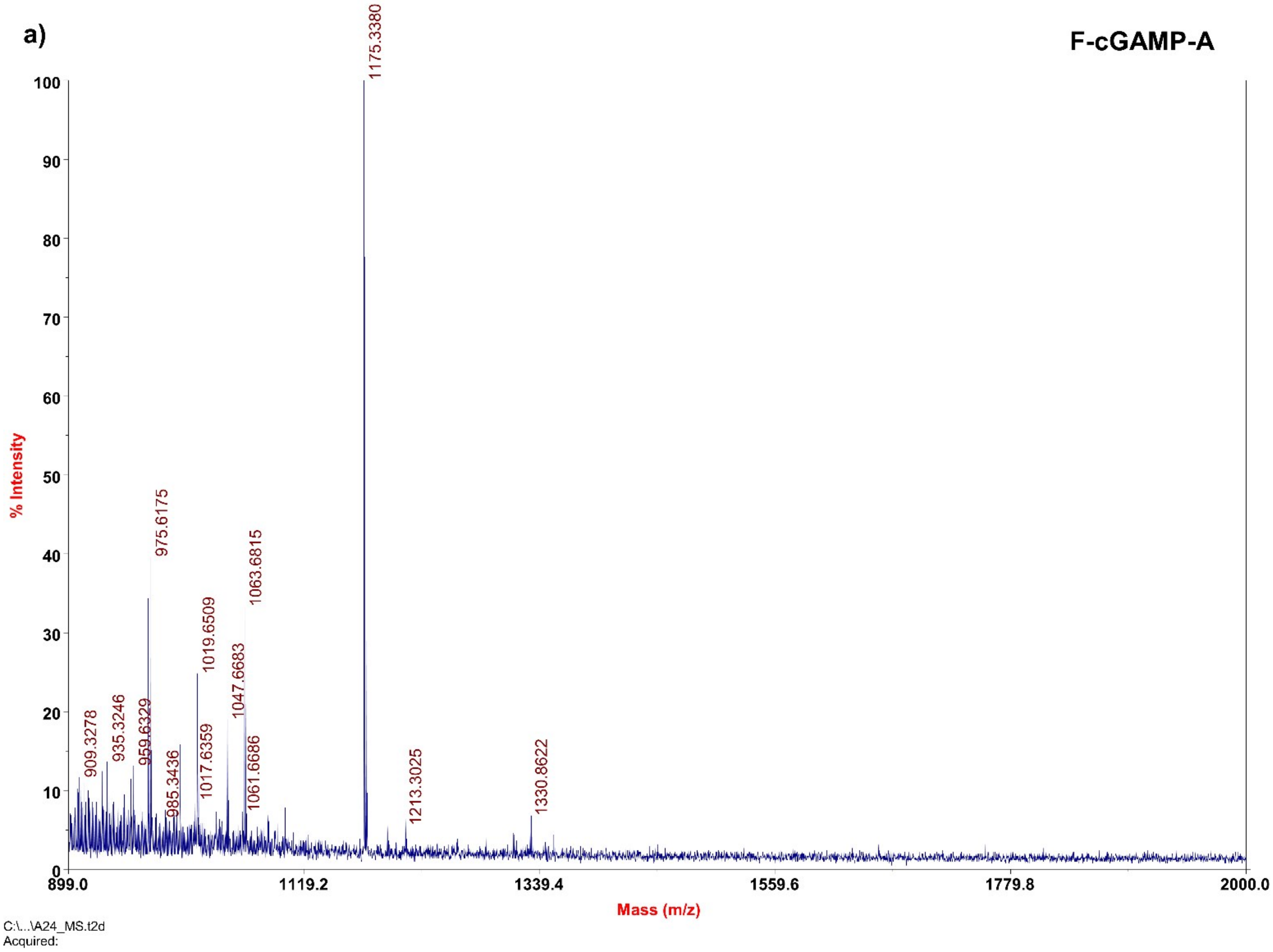
F-cGAMP-A
1175.3380
975.6175
1063.6815
1019.6509
1047.6683
909.3278
935.3246
959.6329
985.3436
1017.6359
1061.6686
1213.3025
1330.8622
% Intensity
Mass (m/z)
899.0
1119.2
1339.4
1559.6
1779.8
2000.0
C:\...\A24_MS.t2d
Acquired:

b)

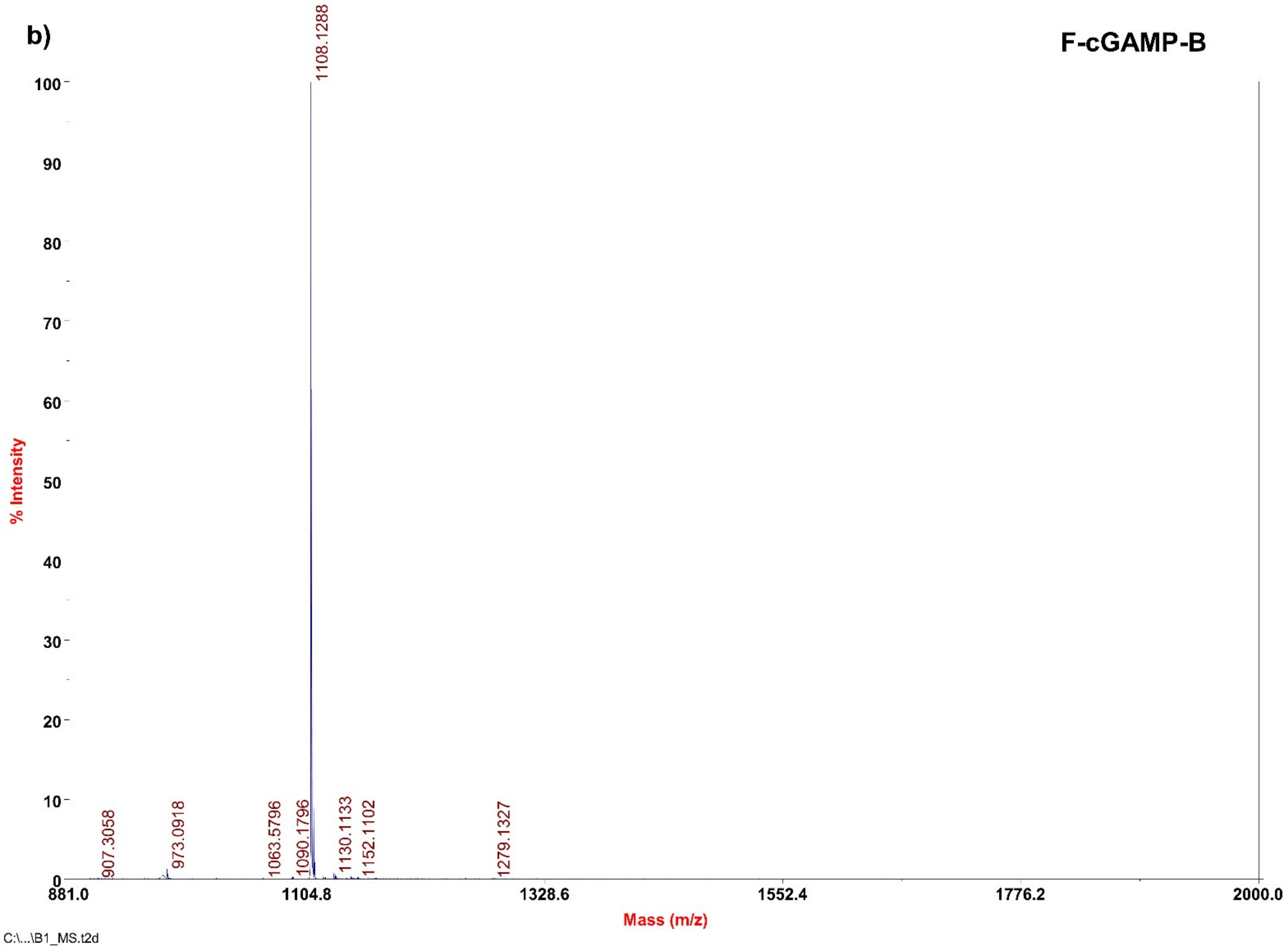

C:\...\B1_MS.t2d
Acquired:

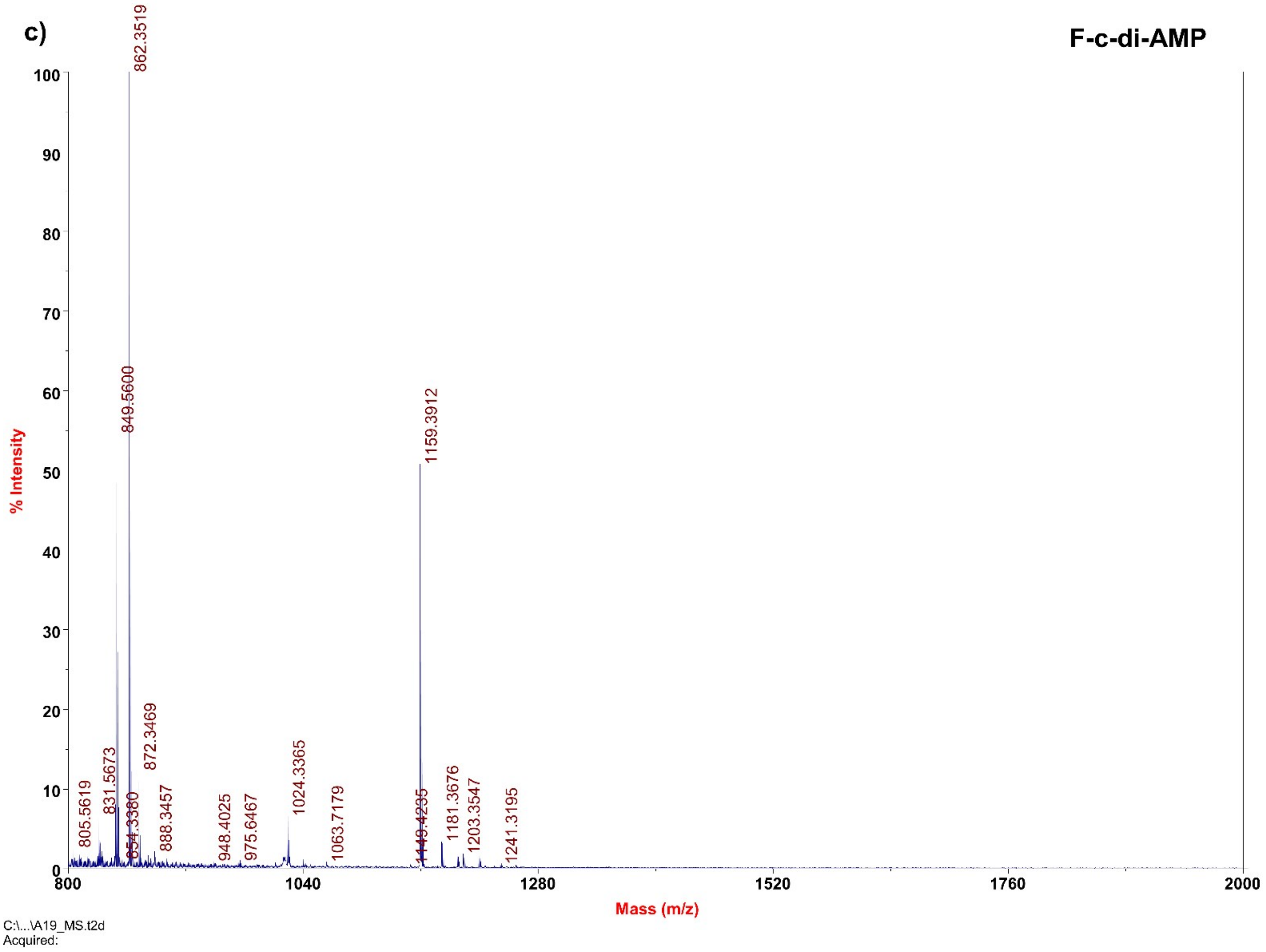
c)
F-c-di-AMP
862.3519
849.5600
1159.3912
872.3469
831.5673
805.5619
854.3380
888.3457
948.4025
975.6467
1024.3365
1063.7179
1149.4235
1181.3676
1203.3547
1241.3195
% Intensity
Mass (m/z)
100
90
80
70
60
50
40
30
20
10
0
800
1040
1280
1520
1760
2000
C:\...\A19_MS.t2d
Acquired:

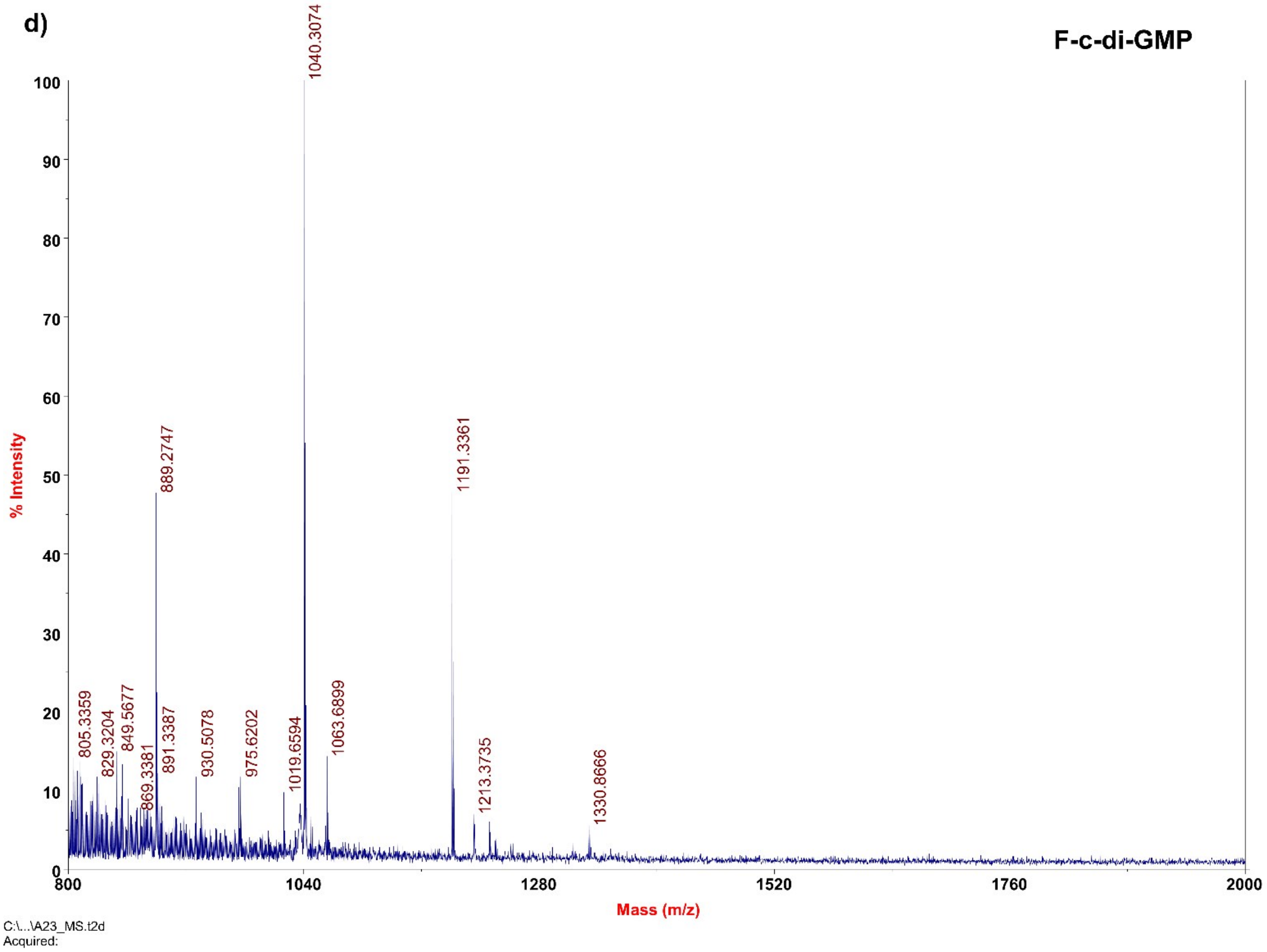

Fig. S6. MALDI-TOF Spectra of Fluorescent cyclic dinucleotides used for the fluorescence polarization assay. a) MALDI-TOF spectra of F-cGAMP-A. Manufacturer reported mass: 1174.9. Found: 1175.3380 ($M+H^+$); b) MALDI-TOF spectra of F-cGAMP-B. Manufacturer reported mass: 1107.9. Found: 1108.1288 ($M+H^+$); c) MALDI-TOF spectra of F-c-di-AMP. Manufacturer reported mass: 1158.9. Found: 1159.3912 ($M+H^+$); d) MALDI-TOF spectra of F-c-di-GMP. Manufacturer reported mass: 1190.9. Found: 1191.3361 ($M+H^+$).
